# Establishment and study of a rat internal haemorrhoid model

**DOI:** 10.1038/s41598-023-48677-2

**Published:** 2023-12-04

**Authors:** Minhui Ke, Shuyan Huang, Haorong Lin, Zhenguo Xu, Xueyu Li, Zuanfang Li, Feng Chen, Huasong Wu

**Affiliations:** 1 Anorectal Department, The Second People’s Hospital Affiliated With Fujian University of Traditional Chinese Medicine, No. 282, Wusi Road, Gulou District, Fuzhou, 350003 Fujian China; 2 Fujian University of Traditional Chinese Medicine, Fuzhou, 350122 Fujian China; 3 The First Hospital of Fuzhou Affiliated With Fujian Medical University, Fuzhou, 350009 Fujian China; 4 Fujian Academy of Chinese Medical Sciences, Fuzhou, 350003 Fujian China

**Keywords:** Diseases, Pathogenesis, Signs and symptoms

## Abstract

To establish a relatively stable internal haemorrhoid model in rats. A total of 48 SPF SD rats were selected and randomly divided into a blank group of 16 and a model group of 32. The model was created by croton oil-mixed liquid stimulation combined with standing and swimming experiments, and the modelling times were 1 week and 2 weeks, respectively. By observing the symptoms and signs of rats, pathological morphology and immunohistochemical staining of anorectal tissue, anorectal laser speckle blood-flow imaging and defecation contrast, etc., the effect of different modelling times was evaluated. The stability of the model was evaluated after feeding for 2 weeks. Both model-formation times caused rats to produce local symptoms of tissue bulging in the haemorrhoid area. Microscopy showed that the rectal submucosal interstitial blood vessels were dilated, and inflammatory cell infiltration and other manifestations were observed. Laser speckle blood-flow imaging revealed increased anorectal blood perfusion and capillary dilatation, and defecography showed a longitudinal and continuous rectal mucosa. After 2 weeks of normal feeding, lifting of the haemorrhoidal tissue was still present. The effect of modelling for 1 week was most in line with the clinical manifestations of internal haemorrhoids. The 1-week modelling scheme in this study can effectively establish a rat internal haemorrhoid model that closely approximates clinical internal haemorrhoid symptoms and pathological manifestations. The operation is simple, the success rate is high, and the model has certain stability. This model can be used as an important basis for studying various treatment methods for internal haemorrhoids.

## Introduction

Conservative drug treatments are often used clinically for early internal haemorrhoids^1^. The establishment of a stable and reliable animal internal haemorrhoid model plays an important role in the development and research of new internal haemorrhoid drugs or the evaluation of the principles and curative effects of internal haemorrhoid drugs. At present, there are few domestic and foreign studies on the establishment of stable and feasible animal models of internal haemorrhoids; most of these studies are aimed at simulating the pathological characteristics^2, 3^, and there are cases where the pathogenic factors are relatively few and the pathogenesis of haemorrhoids cannot be completely replicated. The purpose of this study is to establish a suitable rat model of internal hemorrhoids, to make a good pre-basis for further study of internal hemorrhoids, and to gradually improve the study of animal models of internal hemorrhoids, with a view to providing an animal model for clinical treatment of internal hemorrhoids. Through a series of practices and explorations, the author established a rat model of internal haemorrhoids by adding methods such as standing and swimming to simulate the rat model of acute haemorrhoid attack with a croton oil mixture^4^. The research report is as follows:

## Materials and methods

### Experimental animals and grouping

Forty-eight healthy SPF grade 8-week-old SD rats, male, weighing 180–220 g, were purchased from Beijing Huafukang Biotechnology Co., Ltd., licence number: SCXK (Beijing) 2019-0008. Experiments were carried out after 1 week of acclimatization. The 48 rats were randomly divided into two groups: (A) blank group: 16 rats, 8 of which were fed normally for 1 week (A1), and 8 of which were fed normally for 2 weeks (A2). Modelling group: 32 rats, including 8 rats treated for 1 week (B), 8 rats treated for 2 weeks (C), 8 rats with treatment for 1 week and normal feeding for 2 weeks (D) and 8 rats with treatment for 1 week and normal feeding for 2 weeks (E). The treatment of the rats complied with the "Guiding Opinions on the Kindness of Experimental Animals"^5^.

#### The blank group

The blank group was subjected to the following: daily lighting: 12 h, temperature: 22–25 °C, relative humidity: 40–60%, free diet, normal feeding.

#### Modelling group

The modelling group was based on the blank group. Models were made according to the following scheme, and the normal feeding method after modelling was the same as that of the blank group.

1. Dilation Using inhalation isoflurane anaesthesia (induction anaesthesia concentration: 3–3.5%, continuous anaesthesia: 1.5–2.5%), the rats were fixed on a homemade fixation frame in the supine position, the anus was fully exposed. After sterilization, tissue scissors were used to cut approximately 0.5 cm longitudinally into the canal sulcus (located at 12 o'clock at the lithotomy position), the inner and 12-point tooth lines, the outer and the outer edge of the sulcus, to the degree of unobstructed drainage. After expansion, the rats were fed normally for 3 days, and a light yellow ulcer at the 12-point incision was visible, with no obvious oedema. The experiment continued after no bleeding was observed.
2. Croton oil stimulation: Distilled water, pyridine, ether and 6% croton oil were mixed at a ratio of 1:4:5:10 to form a croton oil mixture^2^. Then, 0.05 mL of croton oil mixture was dropped into the mucosa on the rat's tooth line with a pipette, and after immobilizing the rat for 10 s, the perianal area was massaged with fingers for 10 s, and the rat was put back into the cage 3 times.
3. Standing experiment At a temperature of 18–20 °C, a plastic cylinder close to the abdominal circumference of the rat and a length of approximately 3 times the body length of the rat was selected, and a plastic cylinder with a diameter of 5 cm and a length of 35 cm was prepared. The rat was placed head up and feet down into the plastic cylinder, and the plastic cylinder was vertically fixed after the feet were exposed so that the rat stood stationary for 6 h.
4. Swimming experiment: the standing rats were placed into warm water at 20–25 °C for 1 h. After 1 h, the rats showed sparse hair and wisps, a lack of energy, unresponsiveness to external stimuli, weak hind legs, trembling, exhaustion and fatigue. The rats were placed in a room with a room temperature of 25 °C to dry their fur for 1 h, and step (2) was repeated after an interval of 6 h.

### Experimental drugs and reagents

The experimental drugs and reagents included 6% croton oil (Beijing Huawei Ruike Chemical Co., Ltd.); pyridine (Beijing Lamboride Trading Co., Ltd.); ether (Xilong Science Co., Ltd.); isoflurane for inhalation (Baxter Healthcare Co.); 4% neutral formaldehyde fixative; kit (SYSMEX company, batch number: R0503, R0508, A0013, A0042, A0062); immunohistochemical reagents (Beijing Bioss Xiong, CD3:bs-10498R, CD34:bs-8996R, CD68:bs-20403R, VEGF: bs-1665R).

### Experimental equipment

The experimental devices were as follows: BIOHIT pipette gun 20–200 μL; microtome (MICROM MH325); pusher (Leica); embedding machine (Leica EG1150H); Olympus BX45 optical microscope; microscope collection head (LG500); laser speckle blood flow imaging system (Pericam PSiSystem 2.0); single-arm brain stereotaxic instrument (Shenzhen Ruiwode Life Technology Co., Ltd.); small animal anaesthesia machine (Shenzhen Ruiwode Life Technology Co., Ltd.); Siemens tablet gastrointestinal AXIOM Luminos dRF; XE-5000 automatic blood cell analyser (SYSMEX Co., Ltd.); BS-420 Mindray automatic biochemical analyser (Shenzhen Mindray Biomedical).

### Material collection and inspection time

All groups of rats were collected on the last day of the corresponding study period, and the materials were taken for pathological observation and safety observation. On the first day of the experiment, each group underwent faecal contrast examination and a laser speckle blood-flow imaging system before taking materials.

### Statistical methods

Statistical SPSS 26.0 was used for data processing and statistical analysis. The Shapiro‒Wilk test was used to check whether the measurement data conformed to the normal distribution. If the measurement data were normally distributed, the t test was used. If the normal distribution was not met, the nonparametric rank sum test was used. P < 0.05 indicated differences that were statistically significant.

### Ethical statement

The study is reported in accordance with ARRIVE guidelines.

### Ethics approval

All procedures involving animals were in compliance with the Ethics Committee of Fujian Academy of Traditional Chinese Medicine, and ethical approval was granted by the ethics review approval number FJATCM-IAEC2018015.

## Observation indicators

All groups of rats were collected on the last day of the corresponding study period, and samples were taken for pathological analyses and safety evaluations. On the first day of the experiment, each group underwent a faecal contrast examination and laser speckle blood-flow imaging before sample collection.

### Symptoms and signs

Referring to the Chinese medicine haemorrhoid animal model preparation specification^6^, combined with the modelling, the weight changes of the rats in each group were recorded. In addition, observations were made as to whether the haemorrhoid prolapsed during defecation, whether the stool was dry, whether there was blood in the stool, and whether there was perianal swelling, ulceration, or mucus in the anus.

### Laser speckle contrast imaging

Using isoflurane for inhalation (induction anaesthesia concentration 3–3.5%, continuous anaesthesia 1.5–2.5%), the rats was fixed on the custom-made fixation frame in a prone position, the part was fully exposed, the rat was analysed by Pericam PSiSystem 2.0, and the region of interest (ROI) was drawn by PIMSoft flow metre imaging software. After the ROI region was drawn, the software calculated the blood flow information of each ROI region, which was used to obtain perianal blood perfusion and observe perianal microangiogenesis.

### Pathological observation

The experimental animals were sacrificed by isoflurane overdose anaesthesia, the liver and kidney tissues were separated with tissue scissors, and the specimens were fixed with 4% neutral formaldehyde fixative. Routine paraffin embedding, sectioning, and haematoxylin–eosin (HE) staining were performed for pathological observation. The anorectum of each rat was separated with tissue scissors, and part of the anorectal tissue was taken along the longitudinal axis of the anus to yield a specimen with a length of 15 mm and a width of 10 mm; the specimen was placed in a specimen box. Routine paraffin embedding, serial sectioning, HE staining, Masson staining and HRP immunohistochemical manual staining (VEGF, CD34, CD68 and CD3) were used for adjacent sections. The pathological and morphological changes in the anorectum were observed under a light microscope, and the number of positive cells in 5 random fields of view was counted under a 20 × microscope. The average number was that of cells found to be positive for VEGF, CD34, CD68 and CD3 in this film.

### Defecography

Examination was performed with the Siemens Flat Gastrointestinal AXIOM Luminos dRF device. Thirty minutes before the examination, 10 mL of Kaiser Lu was administered via the anus. During the examination, each rat was fixed on a custom-made fixation frame, 5 mL of gastrogragramine was extracted with a 10 mL syringe and slowly perfused from the anus to the rat's intestine through a 16F suction tube. The faeces passing through the rectum were observed by continuous dynamic shooting. During the filming process, two positions, the left lying position and the prone position, were selected to observe the rat's rectum from different angles.

### Safety observation

After overdose isoflurane was used to anaesthetize the rats, abdominal aortic blood was taken, 2 mL of whole blood was collected, and 3 mL of serum was prepared, of which whole blood was used for the analysis of routine blood and whole blood elements, and serum was used to detect liver and kidney function indices. Routine blood testing items included the white blood cell count, neutrophil count, haemyoglobin concentration, and interleukin 6 level, which were detected by an XE-5000 automatic blood cell analyser. Phosphatase, urea and creatinine were detected by a BS-420 Mindray automatic biochemical analyser.

## Research results

### Symptoms and signs

See Table 1.

**Table 1:** Symptoms and signs of each group.

Group	Prolapse	Constipation	Haematochezia	Tumefaction	Ulcer	Mucus
Blank	No	Obvious	No	No	No	No
Modelling for 1 week	Obvious (split flap prolapse)	More Obvious	More Obvious	Obvious	Obvious	More Obvious
Modelling for 2 weeks	Obvious (annular prolapse)	More Obvious	More Obvious	Obvious	Obvious	More Obvious
Modelling for 1 week; normally reared for 2 weeks	More Obvious (split flap prolapse)	Obvious	Obvious	Apparent	Apparent	Apparent
Modelling for 2 weeks; normally reared for 2 weeks	More Obvious (annular prolapse)	Obvious	Obvious	Apparent	Apparent	Apparent

### Detection results of laser speckle blood flow imaging

#### Blood flow imaging image

**Figure 1:**
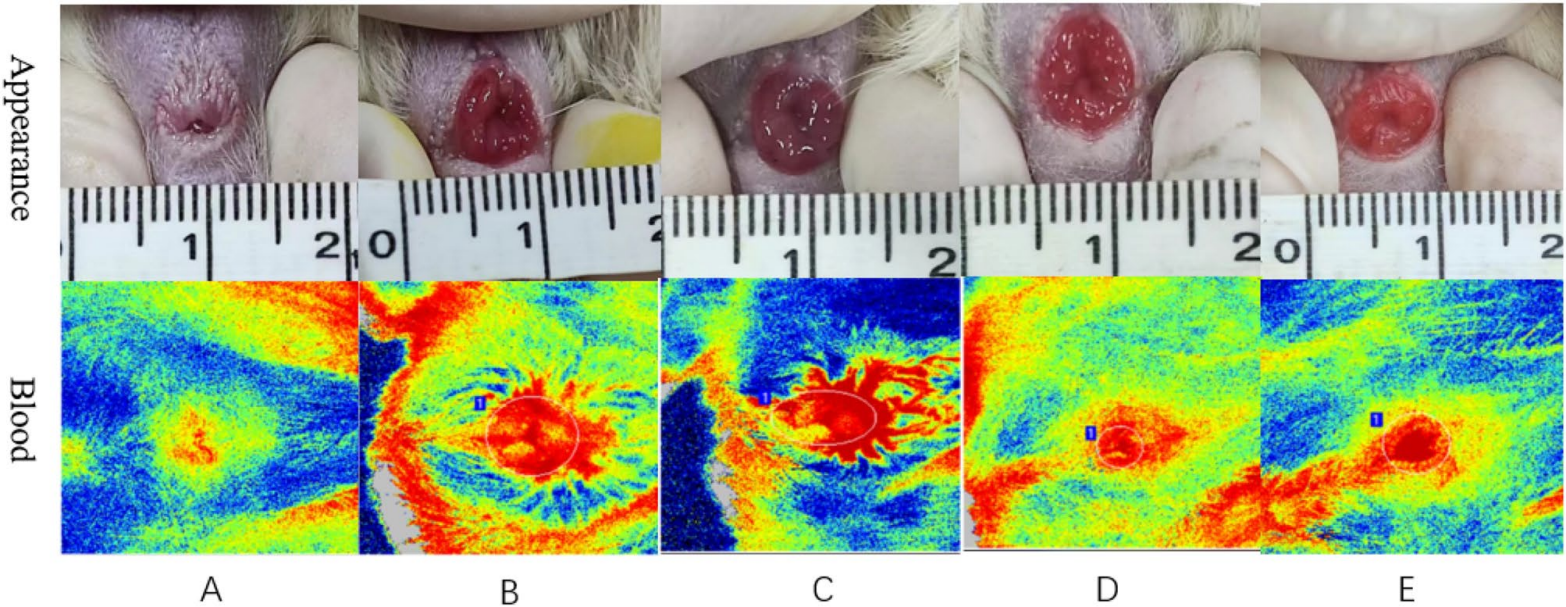
Appearance and blood flow imaging of rats in each group (A: blank, B: modelling for 1 week, C: modelling for 2 weeks, D: modelling for 1 week; normally reared for 2 weeks, E: modelling for 2 weeks; normally reared for 2 weeks).

The blood flow images produced by laser speckle blood-flow imaging (Fig. 1) showed that there was no dilation of perianal microvessels in the blank group. In the modelling group, as the modelling time increased, the perianal microvessels dilated significantly, and branched poplar new blood vessels could be seen 2 weeks after modelling. The blood flow images of normal feeding after modelling (Fig. 1D,E) showed that the expansion of perianal microvessels increased more than that of the corresponding modelling after 1 week of modelling and 2 weeks of modelling after normal feeding for two weeks. At 1 week and 2 weeks after modelling, the symptoms and signs were relieved.

#### Perianal blood perfusion

**Figure 2:**
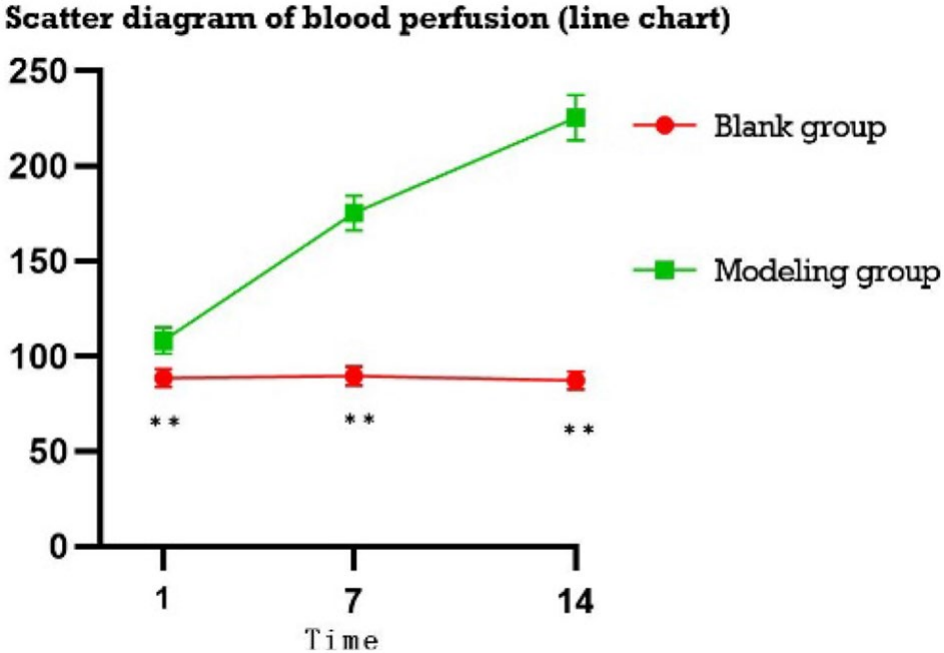
Scatter diagram of blood perfusion (line chart).

**Figure 3:**
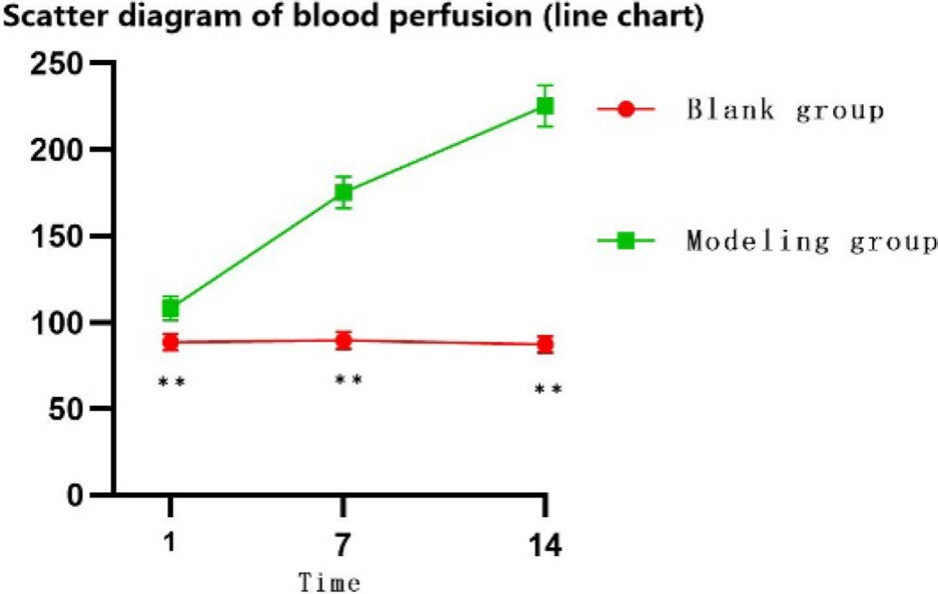
Scatter diagram of the blood flow after normal feeding after modelling (D: modelling for 1 week; normally reared for 2 weeks, E: modelling for 2 weeks; normally reared for 2 weeks).

The perianal blood perfusion of rats detected by laser speckle blood flow imaging was analysed by repeated measures. From Fig. 2, the perianal ROI blood perfusion of the blank group D1. There was no significant difference between D7 and D14 (P > 0.1), while the perianal ROI blood perfusion increased significantly in the model-making group as the model-making time increased (P < 0.05). As shown in Fig. 3, after 2 weeks of normal feeding after modelling for 1 week and 2 weeks of modelling, compared with the corresponding groups with modelling for 1 week and 2 weeks, the blood perfusion of the ROI was significantly reduced, and this difference was significant (P < 0.05).

### Pathological observation

**Figure 4:**
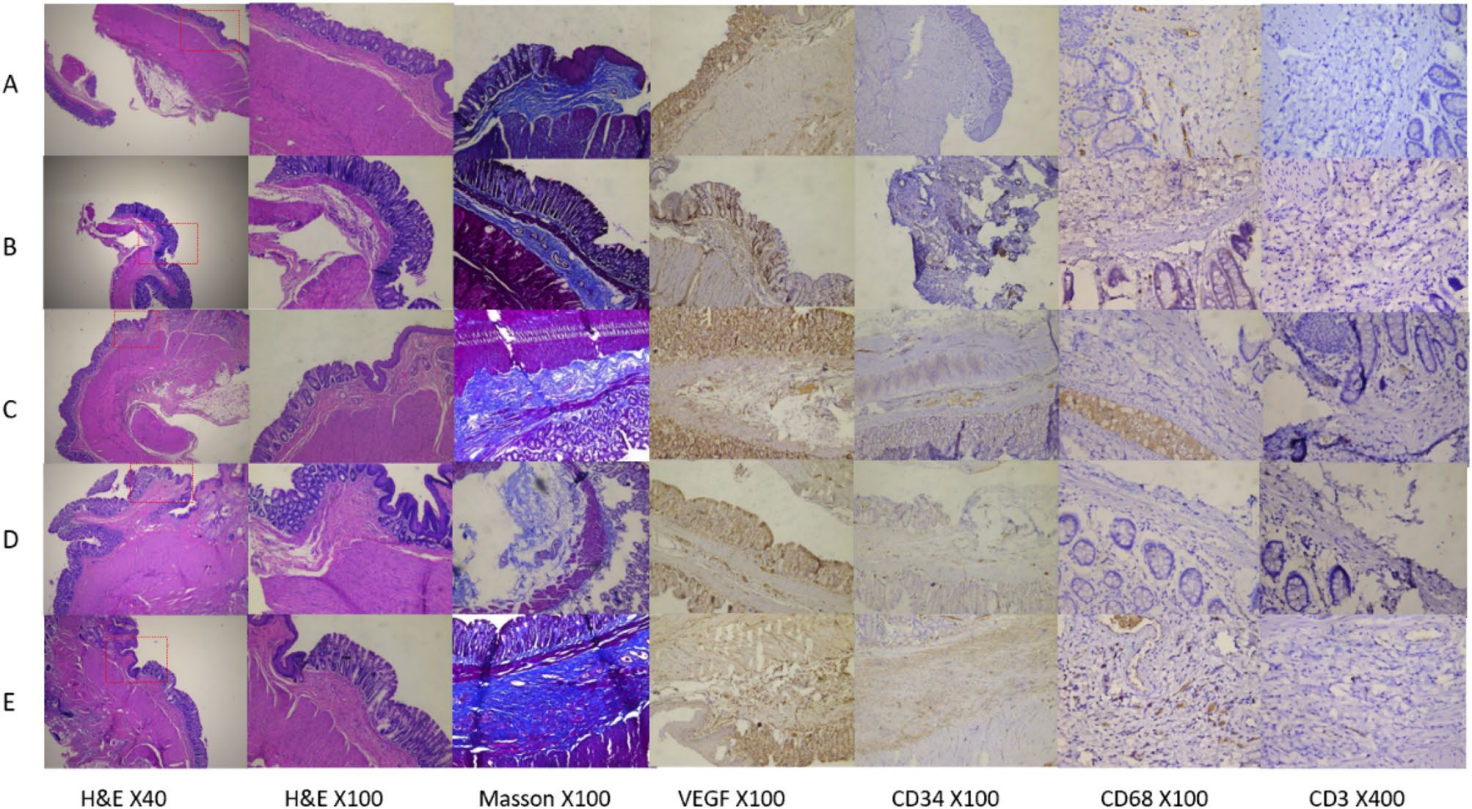
Microscopic appearance of the haemorrhoid areas of the rats in each group.

**Figure 5:**
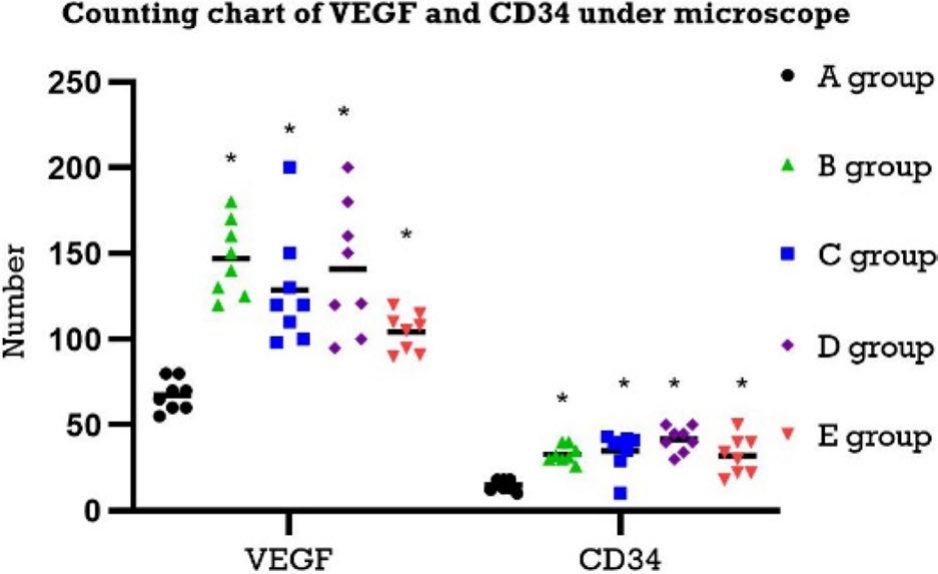
Counting chart of VEGF and CD34 under microscope (A: blank group, B: modelling for 1 week group, C: modelling for 2 weeks group, D: modelling for 1 week; normally reared for 2 weeks, E: modelling for 2 weeks; normally reared for 2 weeks).

The scatter diagram of VEGF and CD34 (Figs. 4, 5) shows that the number of VEGF- and CD34-positive cells in the blank group was less than that in the model group, and the difference was significant (P < 0.05), but in the 1-week model group and the 2-week group, there was no significant difference in the number of VEGF- and CD34-positive cells under the microscope between 1 week of normal feeding and 2 weeks of normal feeding (P > 0.05).

**Figure 6:**
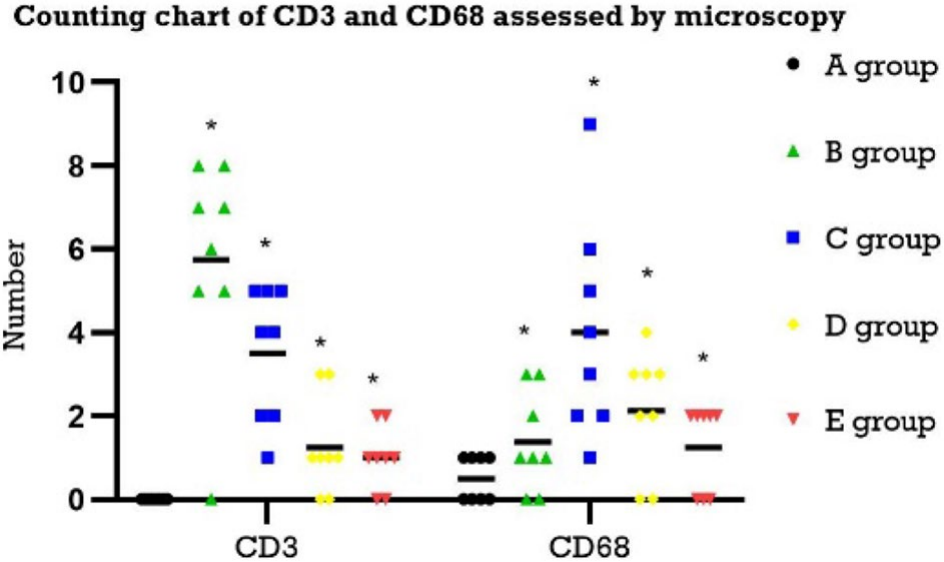
Counting chart of CD3 and CD68 assessed by microscopy (A: blank group, B: modelling for 1 week group, C: modelling for 2 weeks group, D: modelling for 1 week; normally reared for 2 weeks, E: modelling for 2 weeks; normally reared for 2 weeks).

The CD3 and CD68 scatter diagram (Fig. 6) shows that the number of CD34- and CD68-positive cells under the microscope in the blank group was smaller than that of the model group, and the difference was significant (P < 0.05). The number of CD34- and CD68-positive cells in the 1-week group decreased, and this difference was significant (P < 0.05). The model was established for 2 weeks, and the number of CD34- and CD68-positive cells in the normal feeding for 2 weeks group was also lower than that in the modelling for 2 weeks group, and this difference was significant (P < 0.05).

### Defecography

**Figure 7:**
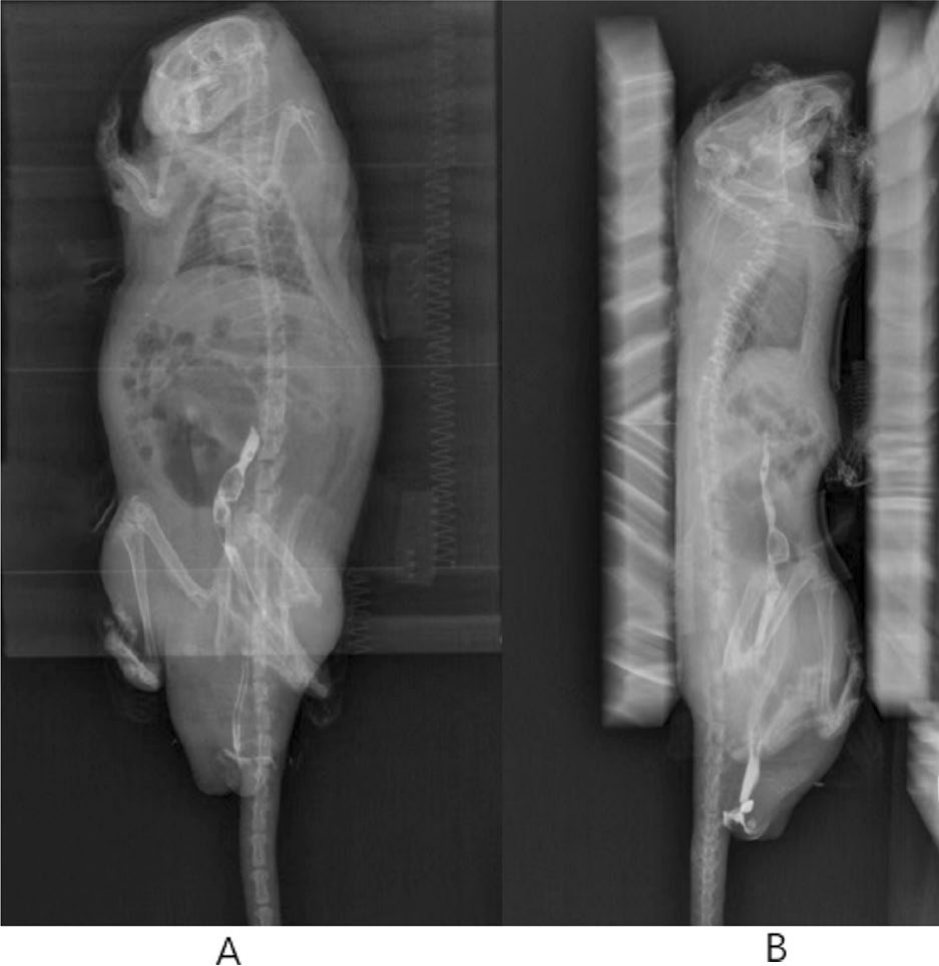
Defecography picture (**A** prone position, **B** lateral position.)

Normal rectal filling, clear contours, natural soft peristalsis, smooth barium passage, a smooth and continuous filling phase bowel wall, no dilatation and stenosis, no niche shadow or filling defect, and a longitudinal, continuous mucosal phase colonic mucosa can be considered to rule out rectal prolapse, prolapse, polyps, ulcers and other diseases (Fig. 7A,B).

### Safety testing and adverse reactions

After the end of the study, the rats in each group were tested for routine blood tests and liver and kidney function, and no abnormalities were found in the test results. The liver pathology of rats in each group showed a clear and complete structure of the liver lobes without degeneration and necrosis; kidney pathology showed a normal and clear structure of the glomeruli and renal tubules, and no obvious inflammatory cell infiltration. The occurrence of perianal ulcers during the modelling process was considered related to irritation of the skin by the croton oil mixture. No rat deaths occurred during the study.

## Discussion

### Design of the modelling scheme of internal haemorrhoids in rats

Currently, the commonly used animal haemorrhoid models mainly include the croton oil mixture haemorrhoid model, the acetic acid injection pathogenic model, the carrageenan pathogenic model, the bacterial pathogenic model, surgical ligation of the rectal vein, etc.^7–10^, all aimed at simulating the pathological characteristics^6^. The problem is that the pathogenic factors are relatively few, and the pathogenesis of haemorrhoids cannot be completely replicated. Among the abovementioned procedures, the method of locally stimulating the rectal mucosa with an inflammatory agent is the most commonly used. Croton oil is a commonly used inflammatory agent that can stimulate the tissue to release leukotrienes, bradykinin, and prostaglandins and cause tissue inflammation^11^. Therefore, this study was based on the haemorrhoid model involving a croton oil mixture. Digestive tract disease models are commonly used with SD rats as the research object^12–14^, and the reference draft^6^ sets the parameters of symptom and sign observation, pathological observation, laser speckle blood-flow imaging changes, and stool excretion imaging to evaluate the success and stability of modelling. By observing the pathological pictures after modelling, obvious vasodilation and inflammatory cell infiltration could be seen on the tooth line, so the model created in this study was a rat internal haemorrhoid model. In the preexperiment, our team designed the 1-week modelling group and the 2-week modelling group and observed that the rectal mucosa of the rats showed split flap detachment after 1 week of modelling, while the rectal mucosa of the rats showed ring-like detachment, similar to the appearance of intrarectal mucosal prolapse, after 2 weeks of modelling. Our team prolonged the observation time after stopping the modelling stimulation to evaluate the stability of this model, so we divided the group with 1 week of modelling and 2 weeks of normal feeding into a group with 2 weeks of modelling and a group with 2 weeks of normal feeding.

Because the anal diameter of rats is small, it is not easy to expose the mucosa of the haemorrhoid area; therefore, the anus was expanded before modelling so that the croton oil mixture was evenly distributed on the mucosa of the haemorrhoid area, and a better observation field of view was obtained. In the original experimental method^2^, a cotton ball soaked in the croton oil mixture was placed into the anus of the rat. In actual operation, the cotton ball will absorb the croton oil mixture, which cannot ensure that the liquid stimulates the rectal mucosa evenly; consequently, pipetting is used instead. The gun accurately controls the dose, and the liquid medicine is dripped quantitatively in batches so that the mucous membrane can evenly absorb the croton oil mixture. Haemorrhoids are produced under the stimulation of multiple factors. Haemorrhoidal disease arises under multifactorial stimuli, and the present modelling scheme was designed based on the previous animal modelling experiments of our team^15^ in combination with the pathogenesis of haemorrhoidal disease. Currently, in international rats, exhaustion swimming is considered a classical model for evaluating the antifatigue effect of drugs^16^. Li^17^ and other teams studied interstitial oedema, skeletal muscle fibre deformation, and obvious inflammatory cell infiltration in rats after swimming exercise. Li^18^ and other teams found that after swimming exercise, rats were withered and emaciated and looked tired and weary, and when out of the water, their limbs were weak. A long time elapsed before the rats were able to shake their own fur, their reaction to the outside world also gradually slowed, their eyes appeared lifeless and numb, and swimming exhaustion manifested. Our team’s rabbit rectal mucosal prolapse modelling^15^ experiments revealed that after the rabbits stood for 6 h per day for 2 months, the rabbit rectal mucosa was lax in the lower layer of the rectal mucosa, the hind limbs were exhausted, the ligaments were lax, and the rectal mucosa presented a prolapsed state. In this study, by simulating the principle of haemorrhoidal prolapse caused by muscle weakness and ligament laxity after fatigue in humans, we designed an increased modelling method of standing and swimming in rats.

### Analysis of the feasibility of the modelling scheme from the observation indicators

As shown in Table 1, the rectal mucosa of rats in model D3 was oedematous, bright red, protruding, and exhibited a split flap. In D6, the haemorrhoid mucosa prolapsed during defecation and then gradually merged, appearing similar to rectal mucosal prolapse. As shown by the changes in the anal mucosa during the modelling process, the oedema and prolapse of the mucosa gradually became more serious over time, and the appearance of human haemorrhoids was clearly visible at D6-7 and then remained stable. According to the pathological and histological observations (Table 2) after the rat model was established, rectal vasodilation, capillary hyperplasia, submucosal interstitial loosening, and inflammatory cell infiltration were observed, which were similar to the pathological changes in human haemorrhoids. Compared with the blank group, the blood flow diagrams of the 1-week modelling group and the 2-week modelling group showed that the microvessels of the rats expanded significantly, and the surrounding new blood vessels formed branch-like structures, which reflected the effectiveness of the modelling method. One week after modelling, the mucous membrane prolapsed, resembling clinical internal haemorrhoid. After 2 weeks, the appearance of the mucosa was circular, resembling rectal mucosal prolapse. Therefore, in this study, the modelling period of 1 week was considered the most appropriate. Changes in the haemorrhoidal mucosa were observed 2 weeks after stopping the modelling. The appearance of the normal anal mucosa of the rats was not restored due to the cessation of the modelling. Hyperplasia was relieved, but there was still a significant difference from the blank group. The physical appearance and blood flow diagram indicated that the modelling method has certain stability.

**Table 2:** The microscopic appearance of each group.

	H&E	Masson
Blank group (Fig. 4A)	The mucosal glands are arranged in a single or double layer, the submucosal interstitial vasodilation is not obvious, and short irregular crypts are visible in the canal transition area	Submucosal collagen fibres are loosely arranged
Modelling for 1 week group (Fig. 4B)	The number of mucosal glands increases, the glands can be arranged in multiple layers, the submucosal interstitial blood vessels are dilated, the curvations are obvious, the number of capillaries is increased, and inflammatory cell infiltration is increased	Submucosal collagen fibres are densely arranged
Modelling for 2 weeks group (Fig. 4C)	Submucosal interstitial vasodilation, marked tortuousness, tissue loosening, space enlargement, accompanied by an increase in the number of inflammatory cells, interstitial fibrous muscular hyperplasia, structural relaxation	Under the mucosa, vasodilation, there is obvious tortuosity; the dense arrangement of collagen fibres is increased, with muscle fibres interspersed
Modelling for 1 week; normally reared for 2 weeks (Fig. 4D)	The decrease in vasodilated hyperplasia was less significant than that in the modelling for 1 week group	Similar to the modelling for 1 week group
Modelling for 2 weeks; normally reared for 2 weeks (Fig. 4E)	The decrease in vasodilated hyperplasia was less significant than that in the modelling for 2 weeks group	Similar to the modelling for 2 weeks group

VEGF can increase vascular permeability and vascular regeneration and induce the formation of a new capillary network. At the same time, when VEGF is highly expressed, it will also aggravate tissue oedema^19–21^. Combining Table 2 with Fig. 5, we believe that VEGF was significantly positive in the modelling group, indicating that angiogenesis was obvious after modelling, accompanied by tissue oedema. In the model group, vascular hyperplasia was observed, mainly concentrated in the mucosal layer, and the lumen of blood vessels in the tissue was significantly expanded. The results of the study showed that the model group was marked by CD34, showing mononuclear stromal cells with a brownish yellow positive reaction. These results are consistent with the increased anorectal blood perfusion and increased microvascular dilatation detected by laser speckle flow imaging. The positive expression of CD68 was seen in the modelling group, and its positive expression was also observed 2 weeks after the cessation of modelling. When macrophages are activated, the expression of CD68 will also increase significantly, which can be used as a marker of macrophages, indicating that local macrophages are activated after modelling, and the inflammatory response is obvious^22^. The results showed that the expression of CD3 staining in the cytoplasm of the 1-week modelling group and the 2-week modelling group was brownish yellow, with a weak reaction and a small number. There was no significant change between the 1-week modelling and 2-week normal feeding groups for 2 weeks. CD3 exists on the membrane surface of mature T lymphocytes. Since memory T lymphocytes can generate a stronger immune response again, CD3 is mainly concentrated in necrotic tissue with severe tissue damage^23, 24^. After local lymphocyte activation, there was no significant damage to the tissue.

In terms of animal adverse reactions and safety, the routine blood, liver and kidney functions of the 1-week modelling group and the 2-week modelling group did not change significantly compared to those of the normal group. This modelling method had no obvious toxic effects in rats. Perianal skin ulcers in rats during the modelling process are considered related to skin stimulation by a croton oil mixture. According to the classic two-stage carcinogenic model of DMBA/croton oil-induced mouse skin papilloma^25^, the Ba soybean oil mixture is destructive to the skin.

### Implications of modelling results for the clinic

#### Correlation analysis between internal haemorrhoids and rectal mucosal prolapse

During the experiment, the author found that although the longitudinal and continuous rectal mucosa appeared normal in the defecography examination, after 2 weeks of modelling, the anus of the rats appeared to prolapse in a ring-shaped mucosa, which was seen under HE staining. Tortuous expansion, tissue loosening, and gap expansion were present; Masson staining revealed that the collagen fibres were densely arranged and increased, accompanied by interspersed muscle fibres and tissue relaxation. The author observed rectal area prolapse in the establishment of a rabbit rectal mucosal prolapse model^26^. Proliferation of glandular tissue in the lamina propria, vasodilation and congestion, infiltration and accumulation of interstitial inflammatory cells, interstitial oedema, and looseness were observed. The pathological manifestation of rectal mucosal prolapse in this model was observed after 2 weeks of modelling. Therefore, the author believes that internal haemorrhoids are the early stage of rectal mucosal prolapse, and the clinical treatment of internal haemorrhoids can delay the progression of rectal mucosal prolapse. This finding can provide experimental design ideas and directions for further research on rectal prolapse disease.

#### Discussion on treatment options for haemorrhoids

In the 2-week modelling group, submucosal collagen fibres were densely arranged and increased under microscopic analysis with Masson staining. This shows that during the formation of haemorrhoids, the body undergoes self-repair. Clinically, we often encounter patients with internal haemorrhoids in the early stage. When they go to the doctor, the main symptom is visible blood. Whether such patients require interventions such as sclerotherapy injections is debatable. From the results of this experimental study, it is possible for this type of internal haemorrhoid to heal itself.

The laser speckle blood-flow imaging system (Fig. 1B,C) showed obvious vasodilation and surrounding angiogenesis that formed branch-like bifurcations after modelling, and the counts of VEGF and CD34 in the haemorrhoid area increased significantly after modelling. The expression was still obvious after 2 weeks of normal feeding after modelling. The author believes that because there are many new collateral blood vessels around the internal haemorrhoid tissue and the immunohistochemical indicators suggest that it is not easy for this condition to subside within a certain period, it is necessary to pay attention to new collateral blood vessels in the recurrence of internal haemorrhoids. Internal haemorrhoids cannot be seen, and during treatment, that is, surgical resection, it is necessary to take measures to improve blood flow around the haemorrhoids to reduce their recurrence.

In summary, this study adopted standardized operation procedures, multiple local inductions, and stimulation of anorectal mucosal prolapse and oedema. In addition, the standing test and swimming test consumed the physical strength of the rats. After fatigue, the muscles relaxed and the haemorrhoids prolapsed, thereby imitating the cause of haemorrhoids. Symptoms and pathological changes occurred in the acute attack stage of haemorrhoids; consequently, the model has a uniform and stable effect, providing a good experimental basis for the discussion and study of animal models of internal haemorrhoids.

## Data Availability

The simulation experiment data used to support the findings of this study are available from the corresponding author upon request.
